# The Mediating Role of Emotional Intelligence in the Organisational Commitment and Turnover Intention of Clinical Nurses: A Cross‐Sectional Study

**DOI:** 10.1002/nop2.70113

**Published:** 2025-01-23

**Authors:** Han Lingyu, Li Ruiling, Wang Yiman, Liu Yafeng, Bai Xiaolu

**Affiliations:** ^1^ The First Affiliated Hospital of Henan University Kaifeng Henan Province China; ^2^ School of Nursing and Health Henan University Kaifeng Henan Province China; ^3^ Huaihe Hospital of Henan University Kaifeng City Henan Province China

**Keywords:** clinical nurses, emotional intelligence, mediation, organisational commitment, turnover intention

## Abstract

**Aim:**

To explore the influence of emotional intelligence and organisational commitment (OC) on clinical nurses' turnover intention (TI) and to provide intervention strategies to reduce the turnover rate of nursing staff and maintain the stability of the nursing team.

**Design:**

A cross‐sectional descriptive study was conducted with nurses (*n* = 452) in a tertiary hospital in Kaifeng City, Henan Province, China.

**Methods:**

The project was conducted in July 2023. The data were collected using the organisational commitment scale, Wong and Law emotional intelligence scale and turnover intention scale.

**Results:**

The emotional intelligence score of clinical nurses was (3.07 ± 0.68), organisational commitment scale was (3.04 ± 0.68), and turnover intention scale was (2.70 ± 0.58). Organisational commitment scale and emotional intelligence scores are negatively correlated with turnover intention; the mediating effect of emotional intelligence in organisational commitment and turnover intention scale of clinical nurses is −0.406, and the mediating effect accounts for 49.9%.

**Public Contribution:**

Nurses' organisational commitment can directly predict turnover intention, and emotional intelligence plays a role in mediating the relationship between nurses' organisational commitment and turnover intention. This research makes a substantial contribution to the public and healthcare sector. The findings provide valuable insights for healthcare administrators, policy‐makers and educators, offering evidence‐based strategies to improve nurse retention and enhance the quality of patient care. It is important to note that the research upholds the highest standards of integrity, with a meticulous review process and a dedicated effort to maintain data quality.

## 1 Introduction

Since the beginning of the COVID‐19 pandemic at the end of 2020, the shortage of nursing staff has become increasingly prominent (World Health Organization, 2020). The shortage and wastage of nurses have become an important concern for medical institutions worldwide. The World Health Organization (WHO) human resources strategy report indicates that by 2030, the global demand for health and social care workers will reach 40 million (WHO, 2020). The global nursing manpower shortage situation is becoming increasingly serious and is also a major problem affecting China. The resignation of nursing staff is an important reason for the shortage of workforce as 20.2%–56.1% of nurses have shown a high tendency to leave (Li et al. 2020). The resignation of nurses will not only increase the labour cost of the hospital and exacerbate the shortage of nurses but also lead to a decline in the quality of care and patient satisfaction, which indirectly affects the efficacy of patients' rehabilitation (Nieuwenhuijze et al. 2024). Early identification and appropriate psychological interventions can reduce nurses' willingness to resign (Chang and Lin 2023).

Emotional intelligence refers to the ability of individuals to monitor their own and others' emotions and recognise and use this information to guide their thoughts and behaviours (Xue et al. 2024). Nursing staff undertake high‐intensity interpersonal interactions and technical tasks at work, whereas those with high emotional intelligence can adjust emotional strategies in time, enhance environmental adaptability and implement care measures according to patients' needs (Baker and Vincent 2024). Organisational commitment (OC) refers to the responsibility and obligation of nurses in hospitals and departments—the higher the level of OC, the higher the organisational cohesion (Geremias, Lopes, and Sotomayor 2024). Research (Galanis et al. 2024; Li et al. 2024; Chen et al. 2024) shows that nurses' turnover intention (TI) is the result of multiple social and psychological factors, namely organisational support, job burnout, job satisfaction and so on, and this can have an impact on nurses' TI. However, previous studies have paid more attention to the impact of a single psychosocial factor on nurse TI and have seldom considered the path and mechanism of the combined effect of multiple variables (Foster et al. 2024).

Therefore, in order to fill this research gap, this study investigates whether emotional intelligence has a mediating effect between clinical nurses' OC and TI, aiming to clarify the relationship and mechanism of action among nurses' OC, emotional intelligence and TI and to explore and improve nursing managers' organisation promises and intervention measures to promote nurses' career stability.

Overall, the problem statement for this study is: whether emotional intelligence has a mediating effect between clinical nurses' OC and TI. In this context, the research objectives of this study are: (1) exploring nurses' levels of emotional intelligence, OC and TI; (2) analysing emotional intelligence has a relationship between clinical nurses' OC and TI; (3) validating emotional intelligence has a mediating effect between clinical nurses' OC and TI; (4) exploring and improving nursing managers' organisation promises and intervention measures to promote nurses' career stability. The research hypothesis is: emotional intelligence does not mediate between clinical nurses' OC and TI.

Through the conduct of this study, we expect to gain insights into the mechanisms of emotional intelligence in clinical care and make important contributions to promoting the quality of healthcare services and the stability of nursing teams.

## 2 Literature Review

Emotional Intelligence: The term Emotional Intelligence first originated from the writings of Aristotle, a famous scholar in ancient Greece, which refers to the fact that anger must be for the right purpose and the right time, manner, degree and object must be chosen (Taylor et al. 2004). Until the 1990s, Mayer, an American psychologist, formally proposed the concept of emotional intelligence and defined it as the ability of individuals to recognise and use emotional information to guide their thoughts and behaviours, pointing out that emotional intelligence consists of three aspects: the ability to assess and express emotions, the ability to regulate emotions and the ability to use emotional information (Lane 2019). In 1997, Baron (Baron 1998) pointed out that emotional intelligence refers to the sum of a series of emotional reactions and individual personality traits and interpersonal skills that affect the individual in the face of the environment and stress, and in 2004, Mayer (Mayer 2004) categorised emotional intelligence into four generally agreed factors, namely, the ability to perceive emotions, to utilise emotions, to understand the emotions and to manage the emotions. At present, there are two main models for scholars' knowledge and understanding of emotional intelligence, one is the competence emotional intelligence model, represented by Mayer and Salovey; the other is the mixed emotional intelligence model, which is represented by Goleman and Baron. The former emphasises the ability to recognise, process and utilise emotional information, while the latter emphasises the intellectual, psychological, personality and motivational aspects of processing emotional information.

Organisational Commitment (OC): Organisational commitment refers to an individual's identification with and trust in the goals and values of the organisation to which he or she belongs, and the resulting experience of positive emotions. OC is one of the more important employee attitudinal variables that can influence the generation of job performance and was first proposed for the first time in 1960. OC belongs to the category of organisational behaviour, widely used to analyse and explore the differences in the behaviour of employees within the enterprise, as the enterprise's investment in the employee continues to increase, the employee decides whether to continue to stay in the enterprise a contract (Afsar et al. 2018). With the deepening of OC research, OC can play an important role in human resource management, but different scholars' research directions and theories based on them are different (Anvari et al. 2023), which leads to bias in their understanding of OC. Compared with Western scholars, Chinese research on OC is relatively small, started relatively late, and most of the research relies more on foreign theories to study organisational commitment. Chinese scholars (Jinlan et al. 2024) believe that OC is an employee's attitude and that the degree of frankness reflects the employee's psychology towards the organisation, predicts whether the employee is willing to continue to stay in the company and reflects the employee's loyalty towards the organisation. Some Chinese scholars also believe (Luo 2024) that OC belongs to a degree of identification and commitment, and employees are able to invest in the organisation in different ways according to their own situation and efforts. At the same time, employees are also willing to take the corresponding responsibility and fulfil the corresponding obligations as a career. Therefore, based on the above research and state, employees can achieve the simultaneous development of individual and organisation through their own work efforts. From the results of the above domestic and international studies, although there are obvious differences in the findings of different studies on OC, most of the studies link the organisation and the employees, and OC as a bridge between the employees and the enterprise, which plays an important role in the occurrence and development of the enterprise (Feifei, Xiaobing, and Yujin 2023).

Turnover intention: Chinese research on turnover behaviour and turnover intention in the nursing field started relatively late. According to the literature review, relevant studies in China started in 2000, and only in 2005 did the number of large‐scale and wide‐ranging relevant studies gradually increase.

The target population of the survey was gradually refined from general nurses to specialties nurses (such as psychiatry, emergency, ICU, operating nurses, etc.) and nurses of various types of establishment (nurses on staff and contracted nurses and other employed nurses (Saihe et al. 2024; Yizhi et al. 2024)), and relevant studies were carried out for nurses of hospitals of different levels and types (general hospitals, specialised hospitals, military hospitals, etc.). The research methodology is consistent with that of foreign countries, with quantitative studies as the main focus and qualitative studies appearing later (first appeared in 2010) and in fewer numbers. The quantitative research is based on cross‐sectional survey research, which mainly analyses the relevant reasons or influencing factors of leaving or willingness to leave and analyses the existing research that: The most important reasons affecting nurses’ resignation: burnout, job satisfaction, organizational empowerment, Commitment, job risks and conditions, achievement motivation, management organization, hospital policy, salary, work‐family conflict, age, nursing age, and career development planning (Lingzhi et al. 2024).

From the above, it can be seen that although there have been many studies focusing on emotional intelligence, organisational commitment and turnover intention (Mahmoodzadeha, Shah, and Abdulaha 2014). However, they are targeted at the general public and lack specialised research for the nursing field. The clinical nursing environment is special, and nurses have patients' illnesses and family members' anxieties in addition to their own emotions, so more specialised research is needed to explore nursing staff. Second, existing studies have been relatively incomplete in exploring the relationship between emotional intelligence and organisational commitment and turnover intention. Although some studies have found their correlation, the mediating role of emotional intelligence in this relationship has not been sufficiently investigated. Elaborating on the mediating role of emotional intelligence is crucial for comprehensive understanding. Expounding on how emotional intelligence functions as a mediator in this context can shed light on its transformative potential within organisational dynamics.

## 3 Material and Methods

### 3.1 Design and Participant Recruitment

Convenience sampling was used to select 452 clinical nurses working in a tertiary hospital in Kaifeng City, Henan Province, China, in July 2023. The inclusion criteria were registered nurses who had been engaged in clinical nursing work in the current unit for ≥ 3 years and who had given informed consent and voluntary participation in the study. Retired or re‐employed nurses as well as nurses who were not on duty during the investigation period due to maternity leave, sick leave, study abroad, retirement, resignation and so on were excluded.

The sample size of this study was based on the sample size estimation method. The actual sample size was 5–10 times the total number of items in the questionnaire (Cho et al. 2021). The scale used in this study, Wong and Law emotional intelligence scale (WLEIS), has 16 entries, Outcome variable of 6 entries and Organisational commitment of 25 entries, which add up to a total of 47 entries. The sample size of this study was eight times the total number of items. Considering that 15% of questionnaires were invalid, the sample size = (16 + 6 + 25) × 8 × (1 + 15%) = 433 cases. In general structural equation modelling research, the sample size was considered to be 200–500 cases (Wu 2009). The surveyed hospital included a total of 900 nurses. Based on these factors, the final sample size of this study was 500. The final questionnaire for this study was 500 and 452 valid questionnaires were returned.

### 3.2 Data Collection

The local ethics committee approved this study. Owing to the impact of the COVID‐19 epidemic, paper questionnaires were not suitable for use; thus, this study used the ‘Questionnaire Star’ app to send QR codes to the mobile phones of nurses in various departments through the hospital's nursing department. The purpose and significance of this survey were indicated at the beginning of the electronic questionnaire. Further, it was specifically mentioned that the questionnaire had to be filled out anonymously, that only survey results were going to be used in this study and that nurses were required to complete the questionnaire truthfully. The researcher's contact information was placed at the beginning of the questionnaire for the nurses to use if they had any questions. A total of 500 nurses were surveyed, 480 questionnaires were returned, 28 invalid questionnaires were removed, and 452 questionnaires were valid, with a recovery rate of 96.00% and an effective recovery rate of 94.17%. The three questionnaires used in this survey have received copyright permission from the original authors.

### 3.3 Measurements

#### 3.3.1 Mediator Variable

Wong and Law emotional intelligence scale (WLEIS) (Wong and Law 2002). The scale was developed and designed by Hong Kong scholars Wong and Low (2002), and it comprises four dimensions and 16 items, including self‐emotional appraisal (SEA), regulation of emotion (ROE), use of emotion (UOE) and others' emotional appraisal (OEA). It uses a Likert 5‐level scoring method (1 = ‘strongly disagree’, 2 = ‘disagree’, 3 = ‘unsure’, 4 = ‘agree’ and 5 = ‘strongly agree’); the higher the score, the higher the EI level of emotional intelligence. The total Cronbach's α coefficient of the scale in this study was 0.94 and that of each dimension was between 0.73 and 0.85.

#### 3.3.2 Outcome Variable

Turnover intention. The scale was first compiled by Michael (Michaels and Spector 1982) in 1982 and was translated and revised by China's Li Dongrong (Li and Li 2000), and it includes six items in three dimensions. TI I includes items 1 and 6, which represent the possibility of employees leaving their current job; TI II includes items 2 and 3, which represent the motivation of employees to find other jobs; and TI III includes items 4 and 5, the possibility of obtaining external work on behalf of employees. The scale uses the Likert 4‐level scoring method, and the answers ‘never’, ‘rarely’, ‘occasionally’ and ‘often’ are given scores of 1 to 4 points, respectively; the higher the score, the stronger the willingness to resign. The Cronbach's α coefficient of the Chinese version of the scale was 0.873, and the content validity was 67.67%.

#### 3.3.3 Independent Variables

Organisational commitment (Ling, Zhang, and Fang 2000). The scale was translated by Ling Wenshuan using 25 items, including five dimensions (sentiment commitment, normative commitment, ideal commitment, chance commitment and economic commitment [EC]). The scale uses a five‐point Likert 5‐level scoring method; the higher the score, the greater the OC. The Cronbach's α coefficient of the scale was 0.88 and that of each dimension was between 0.67 and 0.85.

### 3.4 Ethical Considerations

The study was approved by the Research Ethics Committee of the Institute. The purposes and methods of the study were explained to all participants. Participation was voluntary. Assurances were provided that responses would be confidential. Those who agreed to participate in the study signed a consent form.

### 3.5 Data Analysis

SPSS 22.0 and AMOS 26.0 statistical software were used to process the data. The measurement data were described by $\bar{x}\pm s$, the relationship between variables was analysed by Pearson correlation, the mediation effect was analysed by multiple linear regression and structural equation model, and *p* < 0.05 was considered as the difference was statistically significant.

## 4 Results

### 4.1 Clinical Nurses' WLEIS, OC and TI Score

The results of this study showed that the WLEIS scores of clinical nurses were (3.07 ± 0.68), and the highest score among the four dimensions was UOE (3.11 ± 0.86). The lowest scores were OEA(3.04 ± 0.89). OC was (3.04 ± 0.68). It was in the middle level, and the SC score (3.09 ± 0.90) was the highest, indicating that nurses' OC mainly comes from their emotional and psychological identification with the hospital, followed by factors such as turnover costs, employment opportunities and obligations.

TI was (2.70 ± 0.58) at a relatively high level (Table 1).

**TABLE 1:** Clinical nurses' WLEIS, OC, and TI scores (*n* = 452).

Variables	$\bar{x}\pm s$
SEA	3.06 ± 0.85
ROE	3.09 ± 0.85
OEA	3.04 ± 0.89
UOE	3.11 ± 0.86
WLEIS	3.07 ± 0.68
SC	3.09 ± 0.90
NC	3.08 ± 0.85
IC	2.97 ± 0.83
CC	3.03 ± 0.84
EC	3.04 ± 0.84
OC	3.04 ± 0.68
TI I	2.71 ± 0.76
TI II	2.71 ± 0.70
TI III	2.67 ± 0.77
TI	2.70 ± 0.58

### 4.2 Correlation Analysis

Correlation analysis showed that OC and WLEIS were positively and negatively correlated with TI, respectively. The higher the clinical nurses' OC and WLEIS scores, the lower their TI, as shown in Table 2.

**TABLE 2:** Correlation of WLEIS, OC, and TI of clinical nurses (*r*).

	SEA	ROE	OEA	UOE	SC	NC	IC	CC	EC	TI I	TI II	TI III
SEA	1											
ROE	0.510**	1										
OEA	0.562**	0.483**	1									
UOE	0.534**	0.423**	0.481**	1								
SC	0.432**	0.410**	0.378**	0.316**	1							
NC	0.332**	0.373**	0.337**	0.266**	0.557**	1						
IC	0.398**	0.380**	0.368**	0.276**	0.554**	0.535**	1					
CC	0.354**	0.356**	0.312**	0.287**	0.513**	0.495**	0.489**	1				
EC	0.354**	0.356**	0.312**	0.287**	0.513**	0.495**	0.489**	1.000**	1			
TI I	−0.443**	−0.454**	−0.418**	−0.423**	−0.439**	−0.396**	−0.354**	−0.351**	−0.351**	1		
TI II	−0.389**	−0.356**	−0.393**	−0.371**	−0.366**	−0.345**	−0.361**	−0.345**	−0.345**	0.384**	1	
TI III	−0.403**	−0.410**	−0.386**	−0.297**	−0.419**	−0.406**	−0.402**	−0.365**	−0.365**	0.425**	0.427**	1

*Note:* **p < 0.001.

### 4.3 Mediating Effect Analysis

According to Wen Zhonglin (Wen et al. 2004) mediating effect law tests the mediating effect of emotional intelligence. OC was used as the independent variable X, WLEIS as the intermediate variable M and TI as the dependent variable for regression analysis. The table shows that the standardised coefficient of X to Y is −0.615, and the coefficient is significant at the level of 0.001. In the second step, the standardised coefficient of X to M is 0.569, and the coefficient is significant at the level of 0.001. After controlling for X, the standardised coefficient of the intermediary variable M to the dependent intermediate variable M, and TI as the dependent variable for regression analysis. The table shows that the standardised coefficient of X to Y is −0.615, and the coefficient is significant at the level of 0.001. In the second step, the standardised coefficient of X to M is 0.569, and the coefficient is significant at the level of 0.001. After controlling for X, the standardised coefficient of the intermediary variable M to the dependent variable Y is −0.432. According to the stepwise test of the total intermediary effect of the intermediary variable, first, the coefficient C is significant; in the second step, the coefficient a and the coefficient b are significant, and the indirect effect is thus significant; in the third step, the coefficient b is significant, indicating that WLEIS plays a part in mediating the relationship between OC and TI, as shown in Table 3.

**TABLE 3:** Analysis of the mediating effect of WLELS on the impact of OC on TI.

Step	Independent variable	Dependent variable	Standardisation factor	*t*	*F*	*R* ^*2*^
First step	OC	TI	c = −0.615	−16.542**	273.624***	0.378
Second step	OC	WLEIS	a = 0.569	14.667***	215.123***	0.323
Third step	OC	TI	C′ = ‐0.369	−9.143***	228.409***	0.504
	WLEIS		b = −0.432	−10.691***		

*Note:* ***p < 0.001, **p < 0.01.

### 4.4 Mediation and Structural Model

**FIGURE 1:**
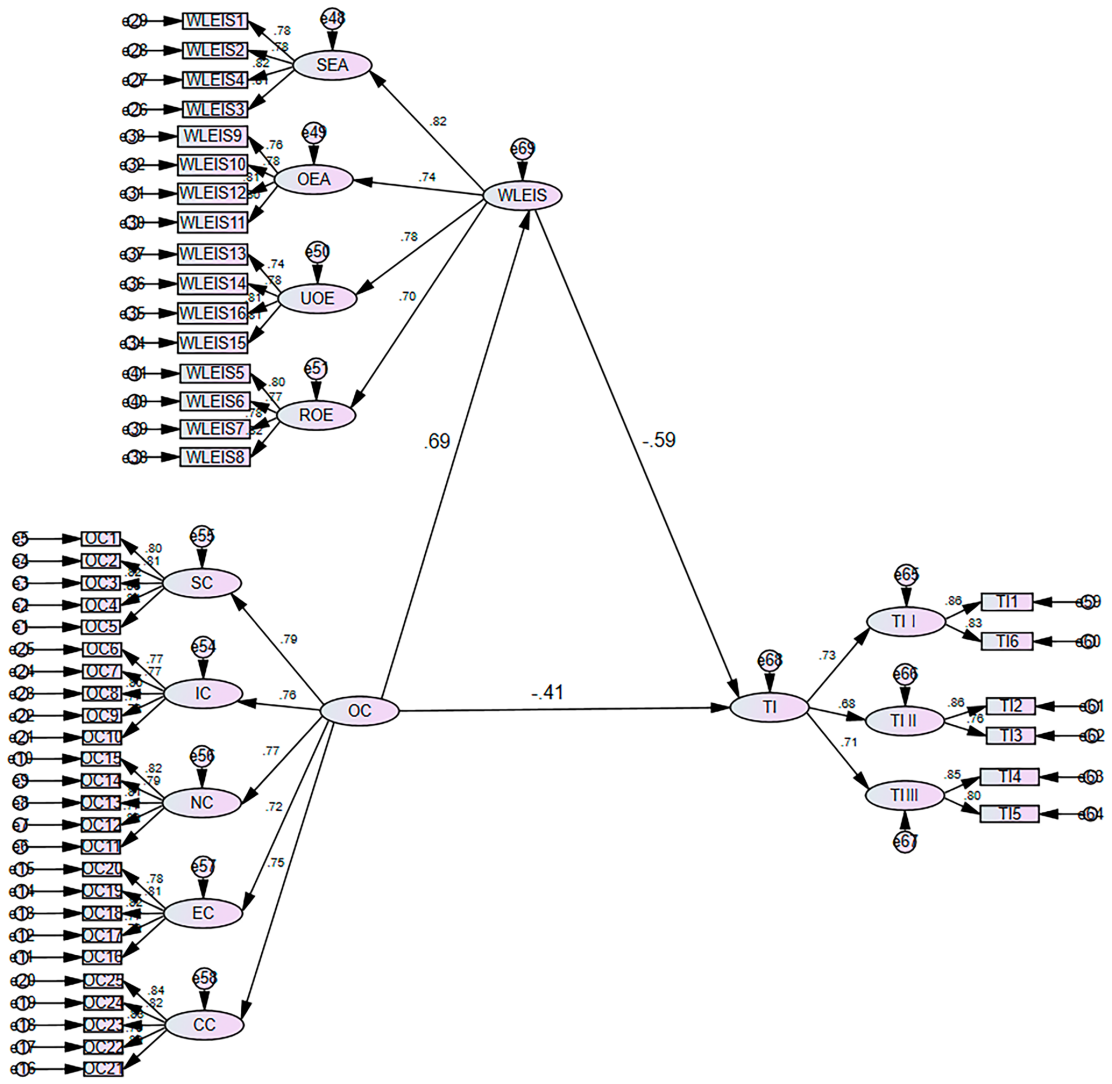
The relationship model of WLEIS in the OC and TI of clinical nurses.

AMOS23.0 software was used to build a structural equation model of nurses' WLEIS, OC and TI (Figure 1), and the maximum likelihood ratio method was adopted to modify and fit the model to verify the hypothesis and then modify the hypothetical model according to the model modification index. All the fitting indices met the requirements. The value of *X* ^2^/df is less than 3; RMSEA and RMR are less than 0.1; and CFI, TLI, NFI and GFI are all greater than 0.9, which is within an acceptable range. Overall, the structural equation model fits well as shown in Table 4.

**TABLE 4:** Evaluation index system and fitting results of the structural equation model's overall fitness.

Fitting index		*X* ^2^/df	RMR	RMSEA	CFI	TLI	NFI	GFI
Initial model		1.197	0.035	0.021	0.985	0.984	0.913	0.9
Fit	Good	< 3.0	< 0.08	< 0.08	> 0.90	> 0.90	> 0.90	> 0.90
Standard	Standard value	3.0–5.0	0.08–0.10	0.08–0.10	0.70–0.90	0.70–0.90	0.70–0.90	0.70–0.90

According to the bootstrap method, the confidence interval of the path OC‐ > WLEIS‐ > TI does not contain 0, indicating that WLEIS can mediate the influence of OC on TI. The mediation effect is −0.406, accounting for 49.9%, as listed in Table 5 and Figure 1.

**TABLE 5:** Path analysis of structural equation model.

Path			Standardisation factor	Unstandardised coefficient	SE	CR	*p*
WLEIS	<−	OC	0.691	0.692	0.067	10.265	M < 0.001
TI	<−	WLEIS	−0.588	−0.447	0.065	−6.926	< 0.001
TI	<−	OC	−0.409	−0.311	0.059	−5.322	< 0.001

## 5 Discussion

### 5.1 Current Status of WLEIS, OC and TI of Clinical Nurses

As shown in Table 1, the WLEIS score of clinical nurses was 3.07 ± 0.68, and the highest score among the four dimensions was UOE (3.11 ± 0.86). This was consistent with the findings of Zhang et al. (Zhang et al. 2012) and Li et al. (Li et al. 2021), and it may be related to the composition of the nursing team. Nurses are mostly women; their emotions are more delicate than those of men, and they are sensitive to changes around them. Perceiving their own negative or positive emotions reminds nurses to learn self‐emotion adjustment, face patients with a positive attitude and use emotion management theory to communicate with patients effectively, reducing patients' anxiety and depression and enhancing nursing care of patients' understanding and trust. The lowest score was OEA (3.04 ± 0.89), which may be due to the heavy clinical work and workload of nurses, who are prone to negative emotions and burnout and thus cannot effectively communicate with patients and their families. The emotional performance and emotional needs of nurses remind nursing managers to organise learning and training on emotional management courses every year to improve their emotional intelligence. When hiring new nurses, nursing managers should not only focus on the assessment of academic qualifications, professional knowledge and operational skills but also on the assessment of emotional intelligence.

This study found that the OC score of clinical nurses was (3.04 ± 0.68), which was in the middle level, and the SC score (3.09 ± 0.90) was the highest, indicating that nurses' OC mainly comes from their emotional and psychological identification with the hospital, followed by factors such as turnover costs, employment opportunities and obligations, which are consistent with the research results of Wei Yan (Yan et al. 2023). In the context of the global shortage and frequent flow of nurses, shaping a democratic and open organisational culture, creating a magnetically attractive organisational atmosphere and further enhancing nurses' sense of belonging and emotional commitment to the organisation have become effective countermeasures for nursing managers to stabilise the nurse team (Gül, Akkaya, and Yildirim 2023).

Table 1 shows that the TI (2.70 ± 0.58) of clinical nurses was at a relatively high level. Since the COVID‐19 outbreak in 2020, nursing staff have had to take the risk of infection at work and wear protective clothing. Changes in the working environment and workflow, high work intensity and irregular work and rest can easily make nurses feel tired and psychologically pressured, producing job burnout (Al Sabei et al. 2023; Gao et al. 2023). The social philosophy of focusing on medical care over nursing, limited career development space and lack of organisational team support can also lead to a decrease in nurses' sense of accomplishment, professional honour, professional well‐being, sense of belonging to the team, lack of enthusiasm for work, feelings of well‐being and feelings of escape (Dorsey 2023). Burnout and negative emotions are important incentives for nurses to leave their job (Quesada‐Puga et al. 2024).

### 5.2 The Relationship Between WLEIS, OC and TI of Clinical Nurses

The model diagram shows that WLEIS has a partial mediation effect between the OC and TI of clinical nurses. The mediation effect was −0.406, accounting for 49.9%; the higher the WLEIS level and OC, the lower the TI of the nursing staff.

OC is an important link that reflects the relationship between employees and organisations. In the field of nursing, emotional commitment refers to nurses' emotional dependence, identification and investment in hospitals: the more the nurse identifies with the hospital, the stronger the emotional dependence and the more dedicated they are to their work. Studies (Bragadóttir et al. 2023) have shown that the longer the working hours, the deeper the nurses' feelings for the organisation and the stronger the sense of dependence; further, the deeper the relationship between the nurse and the hospital, the stronger the willingness to stay. The emotional commitment can give the nurse a strong sense of belonging, and nursing psychological behaviour has a positive role in promoting it; therefore, hospital managers should strengthen communication with nurses, respect and care for them and promote their emotional commitment and engagement in work.

Emotional intelligence is the ability of individuals to effectively manage their own and others' emotions and to use information to control their thoughts and behaviours. Emotional intelligence, as an important soft power in nursing, is not only the professional quality that nurses possess when implementing nursing measures and communication, but it is also indispensable wisdom and ability: the higher the level of emotional intelligence, the better the ability to correctly understand one's own emotions and effectively regulate them in a timely and effective manner, to avoid negative emotions from affecting work and to have stronger psychological endurance when encountering setbacks. Emotional intelligence has a high degree of positive effect on individual ability (Saikia et al. 2024) and plays an important role in individual decision‐making behaviour, leadership and cognitive intelligence. In clinical nursing work, if nurses are not good at regulation, they are prone to emotional disorders, emotional coordination and emotional deviation. Excessive emotional labour backlogs lead to job burnout and exhaustion, which are the biggest threats to the nursing profession. Therefore, in addition to professional skills, an excellent clinical nurse should also have a higher level of emotional intelligence, which helps improve the level of core competence. Studies by foreign scholars (Pandey and Sharma 2024) have shown that emotional intelligence can be improved by nurturing it, and it will remain stable for a long time. Training methods such as environmental intervention and behavioural change can improve the emotional intelligence level of registered nurses. At the same time, individuals with high emotional intelligence may reduce work pressure, accurately convey information, maintain good interpersonal relationships among diverse groups, communicate with others, cooperate and complete organisational goals and ultimately reduce their willingness to leave.

## 6 Conclusion

This study identified the importance of emotional intelligence in explaining clinical nurses' organisational commitment and turnover intention through a cross‐sectional study. The results indicated that emotional intelligence had a positive impact on nurses' organisational commitment and mediated it by reducing turnover intentions. This finding underscores the critical role of emotional intelligence in nursing and provides guidance for future practice, including the development of emotional intelligence training programmes and improving organisational climate. Second, this study provides some theoretical insights. By introducing emotional intelligence into the study of organisational behaviour, we enriched the theoretical framework regarding the relationship between individual traits and organisational outcomes. The addition of emotional intelligence as a mediating variable reveals the mechanism by which individual psychological traits play a role in organisational behaviour and provides new perspectives for the development of organisational behaviour theory. Finally, future research could continue to delve deeper into the relationship between emotional intelligence and nurses' organisational behaviour. For example, the effects of different dimensions of emotional intelligence on organisational commitment and turnover intention could be further investigated, as well as differences across cultures and work environments. In addition, consideration could be given to exploring other potential mediating variables for a comprehensive understanding of the factors influencing nurses' behaviours and organisational outcomes. In summary, this study provides important insights for understanding clinical nurses' organisational behaviours, highlights the critical role of emotional intelligence in nursing settings and provides directions for future research and practice.

## 7 Implications

This study found that emotional intelligence plays a mediating role between nurses' organizational commitment and turnover intentions, which suggests that managers can indirectly enhance nurses' organizational commitment and reduce turnover tendencies through targeted interventions, such as emotional intelligence enhancement programs. This provides practical guidance for hospitals in developing human resource management strategies and emphasizes the importance of building supportive work environments and mental health services. In addition, the results of the study provide a basis for policy makers to recommend policies to promote the construction of nurses' career support system and improve their mental health and career satisfaction. Future studies should incorporate a longitudinal design to delve into the long‐term effects of emotional intelligence and explore the specific mechanisms of diverse interventions on nurses' career stability, thereby further optimizing the nursing management model and enhancing team effectiveness.

## 8 Theoretical and Practical Significance

This study contributes not only at the theoretical level but also in terms of practical applications. First, the identification of emotional intelligence as a mediator helps to reveal the mechanisms underlying the relationship between organisational commitment and turnover intention among clinical nurses. Understanding these mechanisms can assist in the design and implementation of effective interventions aimed at enhancing organisational commitment and reducing turnover. Second, by emphasising the importance of emotional intelligence, healthcare organisations can develop targeted training programmes to develop and enhance the emotional intelligence competencies of clinical nurses, resulting in a more supportive and resilient nursing team. In addition, the findings highlight the importance of creating a positive climate within the organisation that values and supports the development of emotional intelligence. Such initiatives can contribute to increased job satisfaction and retention of nurses and ultimately improve the quality of patient care.

By elucidating the mediating role of emotional intelligence in the relationship between organisational commitment and turnover intention, this study theoretically advances the framework in related areas. Understanding these mechanisms contributes to a comprehensive understanding of the role of emotional intelligence in organisational dynamics, especially in the specific context of clinical care.

At the same time, this study suggests practical strategies for using emotional intelligence to enhance organisational commitment. By incorporating emotional intelligence training and development into organisational policies, healthcare organisations can enhance nurse retention, job satisfaction and overall organisational effectiveness. The practical application of the findings could lead healthcare organisations to develop targeted interventions to reduce turnover intentions and develop a more committed nursing workforce with high emotional intelligence.

## 9 Originality

This study fills a gap in existing research by systematically exploring, for the first time, the effects of emotional intelligence as a mediating variable on organisational commitment and turnover intention in a clinical nursing setting. Through empirical research, we reveal the critical role of emotional intelligence in this specific occupational group, further enriching the application of emotional intelligence theory in nursing. The uniqueness and novelty of this research perspective provide a new theoretical basis and practical guidance for nursing management and human resource management.

## 10 Contributions

It adds to the growing body of literature exploring the role of emotional intelligence in healthcare contexts, particularly within the nursing profession. By empirically demonstrating the mediating effect of emotional intelligence on organisational commitment and turnover intention, this study extends our understanding of the factors influencing nurse retention and job satisfaction. Moreover, by employing a cross‐sectional design, this research provides a snapshot of the current state of emotional intelligence, organisational commitment and turnover intention among clinical nurses, offering valuable insights for targeted interventions. Additionally, the inclusion of emotional intelligence as a mediating variable represents a novel contribution to the literature, highlighting the importance of considering individual‐level factors in explaining organisational behaviour and outcomes.

## 11 Impact

The findings of this study have practical implications for healthcare organisations striving to enhance nurse satisfaction and retention rates. By recognising the pivotal role of emotional intelligence in shaping organisational commitment and turnover intention, healthcare leaders can implement evidence‐based strategies to support emotional intelligence development among clinical nurses. Ultimately, these efforts can contribute to a more engaged and resilient nursing workforce, leading to improved patient care outcomes and organisational performance.

## 12 Research Limitations

This study has some limitations. First, due to the cross‐sectional design, causal relationships cannot be inferred. Second, the samples mainly come from specific regions or types of medical institutions, which limits the generalisability of the research results. In addition, this study only examines emotional intelligence as a mediating variable without considering other possible influencing factors.

## 13 Future Research Directions

Future studies can adopt a longitudinal design to explore causal relationships. Expand the sample range to improve the generalisability of the research results. Consider other influencing factors, such as job satisfaction and work stress, to obtain a more comprehensive understanding. At the same time, further explore the definition and measurement methods of emotional intelligence to deepen the understanding of its role in the organisational behaviour of clinical nurses.

## Author Contributions

Han Lingyu: Writing original draft, Writing review and editing. Li Ruiling: Writing review and editing, Proofreading. Wang Yiman: Supervision, Writing review and editing. Bai Xiaolu, Liu Yafeng: Conceptualisation, Investigation.

## Conflicts of Interest

The authors declare no conflicts of interest.

## Data Availability

The data that support the findings of this study are available from the corresponding author upon reasonable request.
